# Antidepressant Effect of Intermittent Long-Term Systemic Administration of Irisin in Mice

**DOI:** 10.3390/ijms23147596

**Published:** 2022-07-08

**Authors:** Patrizia Pignataro, Manuela Dicarlo, Roberta Zerlotin, Giuseppina Storlino, Angela Oranger, Lorenzo Sanesi, Roberto Lovero, Cinzia Buccoliero, Giorgio Mori, Graziana Colaianni, Silvia Colucci, Maria Grano

**Affiliations:** 1 Department of Basic Medical Sciences, Neuroscience and Sense Organs, University of Bari, 70124 Bari, Italy; manuela.dicarlo@uniba.it (M.D.); giuseppina.storlino@uniba.it (G.S.); lorenzo.sanesi@uniba.it (L.S.); silviaconcetta.colucci@uniba.it (S.C.); 2 Department of Emergency and Organ Transplantation, University of Bari, 70126 Bari, Italy; roberta.zerlotin@uniba.it (R.Z.); angela.oranger@uniba.it (A.O.); cinzia.buccoliero@uniba.it (C.B.); graziana.colaianni@uniba.it (G.C.); maria.grano@uniba.it (M.G.); 3 Clinical Pathology Unit, Polyclinic of Bari, 70124 Bari, Italy; robertolovero69@gmail.com; 4 Department of Clinical and Experimental Medicine, University of Foggia, 71100 Foggia, Italy; giorgio.mori@unifg.it

**Keywords:** depression, antidepressant, irisin, FNDC5, physical exercise, open field test, tail suspension test, forced swim test, neurotrophins, mice

## Abstract

Depression is a psychiatric disorder increasingly diffused worldwide. Evidence suggests that irisin, a myokine secreted by contracting muscle, mediates beneficial effects on several targets, including the brain. Here, the potential antidepressant properties of long-term intermittent systemic irisin administration (100 µg/kg/weekly for 1 month) were evaluated in mice by the Tail Suspension Test (TST), Forced Swim Test (FST), and Open Field Test (OFT). Furthermore, to deepen the molecular pathways underlying irisin treatment, the expression of irisin precursor, neurotrophic/growth factors, and cytokines was analyzed. Irisin treatment significantly decreased the immobility time in the TST and FST, suggesting an antidepressant effect. Additionally, irisin seemed to display an anxiolytic-like effect increasing the time spent in the OFT arena center. These findings were probably due to the modulation of endogenous brain factors as the gene expression of some neurotrophins, such as brain-derived neurotrophic factor (BDNF) and insulin-like growth factor (IGF-1), was upregulated only in irisin-treated mouse brain. Moreover, irisin modulated the expression of some cytokines (IL-1β, IL-4, IL-6, and IL-10). To the best of our knowledge, this is the first study demonstrating that the irisin antidepressant effect may be observed even with a systemic administration in mice. This could pave the way toward intriguing preclinical research in humans.

## 1. Introduction

Clinically significant depression (Major Depressive Disorder, MDD) is a psychiatric disorder characterized by a persistent depressed mood, anhedonia (lack of interest and pleasure), sleep/appetite alterations, impaired cognitive function, and, in the most severe form, recurrent thoughts of suicide or death. Depression represents the most common mental disorder. Indeed, according to the latest available estimates of the World Health Organization (WHO), over 300 million people (about 4.4% of the world population) are estimated to suffer from depression worldwide [1].

Despite its wide distribution, the precise etiology of depression is still unclear; multiple factors, such as genetic, biological, and psychological influences, may play a key role in the onset and/or maintenance of the depressive disease. Recent studies demonstrated that also physical environment features, such as the presence of air chemical pollutants, may increase the incidence of depression in the population [2].

Several theories have been proposed to elucidate the pathophysiological basis of MDD. The most common hypothesis is the dysfunction of monoaminergic neurotransmission, according to which the concentrations of monoamines, such as serotonin, noradrenaline, and dopamine, in the synaptic cleft are decreased in depressive disorders (“monoamine depletion hypothesis”) [3]. In addition to this central topic of depression research, in the last years, the evidence demonstrated the implication of the inflammatory process in a subset of depressed subjects [3]. Some MDD patients displayed higher circulating levels of proinflammatory cytokines, including interleukin 6 (IL-6), tumor necrosis factor-alpha (TNF-α), interleukin 1β (IL-1β), and C-reactive protein (CRP), compared to controls [3,4]. The kynurenine (KYN) pathway has been implicated as a possible mechanism that links the proinflammatory cytokines and the depressive state [5,6]. Indeed, inflammatory mediators activate the KYN pathway in which the tryptophan (TRP), the precursor of serotonin, is degraded into metabolites known as kynurenines. As a consequence, TRP depletion decreased the serotonin level in plasma, increasing the risk of developing depressive symptoms [5]. Furthermore, the metabolic KYN pathway leads to the production of quinolinic acid (QUIN) and 3-hydroxykynurenine (3-HK), which act as neurotoxic factors in the central nervous system (CNS) [5,6]. Besides QUIN and 3-HT, other KYN pathway metabolites displaying a neuroprotective/neurotoxic effect in a concentration-dependent manner (i.e., the kynurenic acid) are under extensive research for their use as potential biomarkers in various psychiatric disorders as well as neurological illnesses, such as migraine [7,8,9,10].

Structural alterations were detected in several brain regions of MDD patients using neuroimaging techniques, such as magnetic resonance imaging (MRI). Significant changes were found in cerebral areas involved in the emotion and cognition functions, two mental processes that are frequently impaired in MDD. Specifically, morphological modifications were observed in the frontal and temporal lobes, the hippocampus, the thalamus, the striatum, and the amygdala [11,12]. Several studies focused on the ventromedial portion of the prefrontal cortex (vmPCF), considering its involvement in fear conditioning, a mechanism that is aberrant in depressive disorders [13,14,15,16]. However, a recent work highlighted the role of the autonomic nervous system in human fear conditioning and its anatomical and functional interplay with the prefrontal cortex [16,17]. An impairment of this network was found in the context of several psychiatric disorders, including depression [16]. 

To shed light on the complex depression pathophysiology, a recent computational psychiatry approach was used to create mathematical models that mimicked the specific neural and/or cognitive functions of depressive behaviors and distinguished them from the anxiety ones [18].

Based on the current knowledge of the disease pathogenesis, antidepressant use and psychotherapeutic interventions represent the actual clinical approaches to reducing depressive symptoms. Nonetheless, these therapeutic strategies are not always effective, as not all depressed patients improve or show a partial response [19].

Over the years, growing evidence has suggested that regular physical exercise is a useful tool for more successful depression treatment in patients with mild to moderate forms of depression, improving mood and promoting psychophysical well-being [20]. Furthermore, it has been demonstrated that physical activity is able to prevent the risk of developing depressive as well as anxiety disorders [20,21]. 

The antidepressant effect of exercise is due to increased production of some mood-regulating neurotransmitters (i.e., endorphins), a reduced release of the stress hormone cortisol, the induction of neurogenesis, and enhanced secretion of neurotrophins. Among them, brain-derived neurotrophic factor (BDNF), nerve growth factor (NGF), insulin-like growth factor (IGF-1), and fibroblast growth factor (FGF-2) improved neuronal survival, proliferation, and maturation [22]. Besides this direct effect of exercise in the CNS, a peripheral mechanism involving skeletal muscle has recently been proposed. During physical activity, the contracting skeletal muscle increases the expression of peroxisome proliferator-activated receptor-gamma coactivator-1alpha (PGC-1α), which, in turn, induces Fibronectin type III Domain-Containing protein 5 (FNDC5) expression [23]. This membrane protein is the precursor of irisin, a newly discovered myokine released into the circulation as a 112-amino-acid polypeptide [23]. Irisin acts as a hormone on several distant targets, including the brain in which its ability to cross the blood–brain barrier has recently been demonstrated by Islam and co-workers [24]. Irisin exerts a beneficial effect on cerebral functions, increasing the expression of BDNF and other neuroprotective genes [25]. Therefore, irisin may modulate cognitive function, memory, learning, and mood [26]. 

The antidepressant properties of irisin have been investigated in a limited number of studies on rodent models. Wang and Pan found that subcutaneous irisin treatment for 14 days suppressed depressive-like behavior in male Sprague–Dawley rats [27]. Similarly, Siteneski et al. demonstrated an antidepressant-like effect of irisin in intracerebroventricular (i.c.v.) injected male mice using behavioral paradigms, such as the Tail Suspension Test (TST) and the Forced Swimming Test (FST) [28]. 

In the present study, the potential antidepressant properties of long-term systemic intraperitoneal (i.p.) irisin administration in young mice was investigated by the combination of different behavioral tests, i.e., TST, FST, and Open Field Test (OFT). Furthermore, to clarify the molecular pathways underlying irisin treatment, the expression of FNDC5 and neurotrophic factors (BDNF, NGF, IGF-1, and FGF-2) was analyzed. Considering that chronic neuroinflammation is a known biological risk factor for depression [3,4,29], the effect of irisin on the expression of selected pro- and anti-inflammatory cytokines (Interleukin-6 (IL-6), Interleukin-1β (IL-1β), Interleukin-10 (IL-10), Interleukin-4 (IL-4), Interleukin-1 receptor antagonist (IL-1ra)) was evaluated.

## 2. Results

### 2.1. Irisin Long-Term Systemic Administration Effect on Mouse Weight

The body weight of the irisin-treated mice and the control group was measured before irisin/vehicle injection. The comparison between the experimental groups showed no differences at each time point, as reported in the supplementary materials (Figure S1).

### 2.2. Irisin Long-Term Systemic Administration Effect on Mouse Behavior in the FST and TST

**Figure 1:**
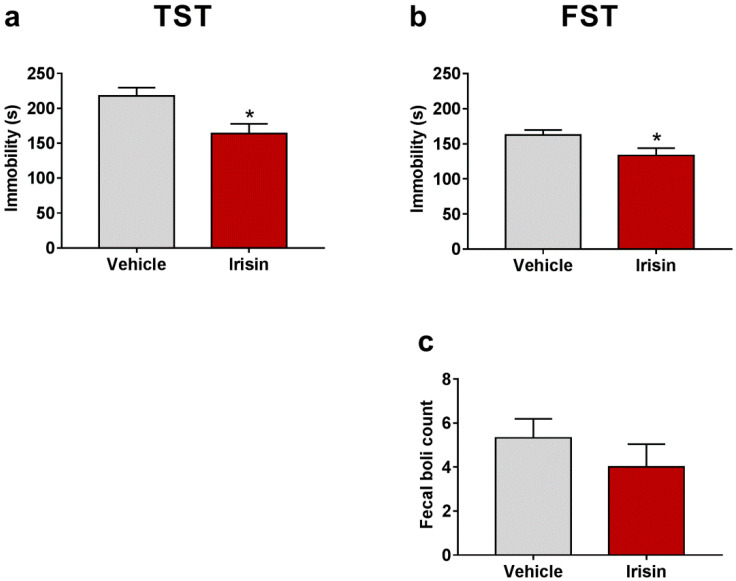
Effect of irisin long-term systemic administration on the immobility time in the TST (**a**) and in the FST (**b**). Number of fecal boli excreted in the FST test (**c**). Histograms represent mean values ± SEM. Unpaired two-tailed Student’s *t*-test was used for statistical analysis. * *p* < 0.05.

The behavioral responses of young mice treated with recombinant irisin (r-irisin) (100 µg/kg/weekly) for 1 month were investigated using the TST and the FST 2 h and 24 h after the last i.p. injection, respectively. The results depicted in Figure 1a,b show that the time spent in immobility is significantly reduced in irisin-treated mice submitted to the TST (163.80 ± 14 vs. 217.80 ± 11.88; * *p* < 0.05) and the FST (133.44 ± 10.39 vs. 162.33 ± 7.409; * *p* < 0.05) compared to the control group. Furthermore, a moderate reduction of fecal boli excretion, not statistically significant, was noted in irisin-treated mice in the FST (4 ± 1.041 vs. 5.33 ± 0.866; *p* = 0.339) (Figure 1c). Table 1 reported a summary of these results.

### 2.3. Irisin Long-Term Systemic Administration Effect on Locomotor Activity in the OFT

**Figure 2:**
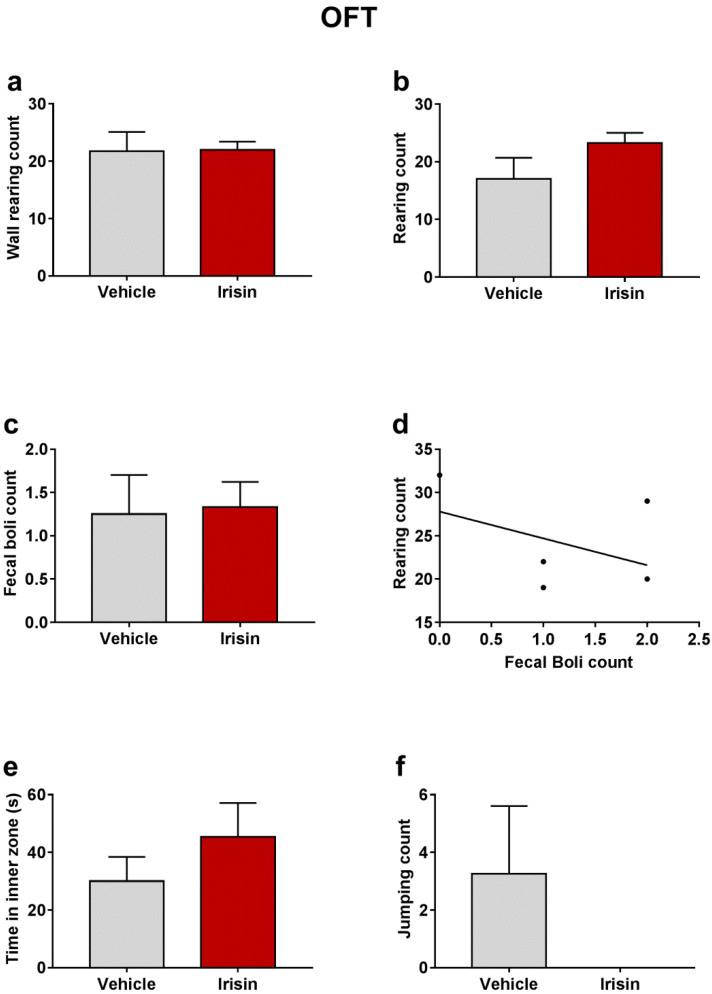
Effect of irisin long-term systemic administration on the wall rearing count (**a**), rearing count (**b**), and fecal boli count (**c**) in the OFT. Correlation between rearing and fecal boli count (**d**). Time spent in inner zone (**e**) and jumping count (**f**) in the OFT. Histograms represent mean values ± SEM. Unpaired two-tailed Student’s *t*-test was used for statistical analysis of wall rearing and rearing count and time spent in inner zone. Unpaired Mann–Whitney test was used for fecal boli and jumping count. The Spearman’s rank correlation test was used to correlate the fecal boli and rearing counts.

In order to exclude the young mice’s locomotor hyperactivity caused by a possible psychostimulant effect of irisin, the OFT was carried out 1 h after the last irisin treatment. Wall rearing counts evidenced that irisin-treated mice showed a similar motor activity as the control group (21.75 ± 3.351 vs. 22 ± 1.414; *p* = 0.9373) (Figure 2a). As far as the rearing behavior is concerned, irisin administration moderately increased the rearing number, even though not statistically significant (23.25 ±1.75 vs. 17 ± 3.769; *p* = 0.1211) (Figure 2b). This observation is indicative of a potential anxiolytic-like effect of irisin. No significant differences were noted in fecal boli number between the two mouse groups (1.25 ± 0.4532 vs. 1.333 ± 0.2887; *p* = 0.8283) (Figure 2c). An inverse correlation was found between rearing and fecal boli counts (ρ = −0.3131, *p* = 0.583) (Figure 2d), and a not significant increase in the time spent in the center of the arena was observed in irisin-treated mice (45.42 ± 11.7 vs. 30 ± 8.426; *p* = 0.3472) (Figure 2e). The number of jumping was also included in the behavioral parameters observed in this test. No jumps were recorded in irisin-treated mice; on the contrary, the control-group mice exhibited a pronounced jumping behavior (0 ± 0 vs. 3.25 ± 2.358; *p* = 0.1333) (Figure 2f). Table 1 reported a summary of these results.

### 2.4. Irisin Effect on Serum Cortisol Levels

**Figure 3:**
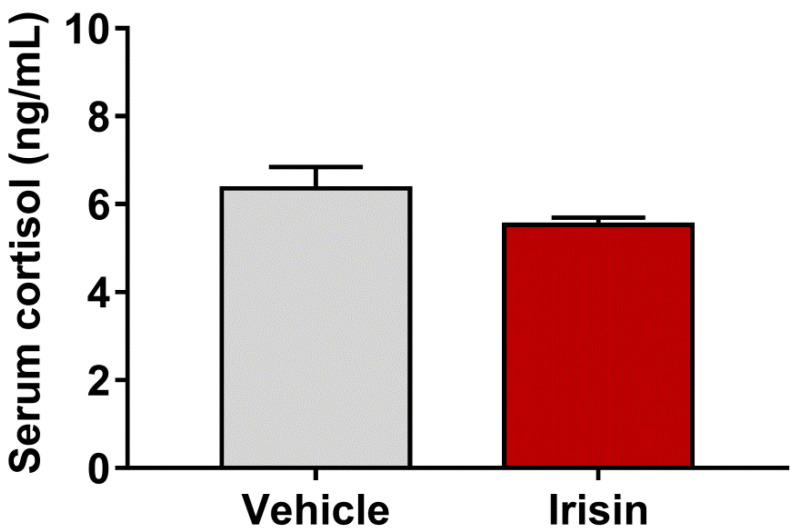
Concentrations of serum cortisol in irisin-treated mice and controls. Histograms represent mean values ± SEM. Results were analyzed with unpaired two-tailed Student’s *t*-test.

Cortisol was detected in the serum of irisin-treated mice and controls, as many studies used this corticosteroid as a stress indicator [30]. A slight reduction, not statistically significant, was observed in cortisol concentrations in irisin-treated mice compared with the control group (5.53 ± 0.1667 vs. 6.361 ± 0.4812; *p* = 0.1554) (Figure 3).

### 2.5. Irisin Long-Term Systemic Administration Effect on FNDC5 and PGC-1α Gene Expression in Brain

**Figure 4:**
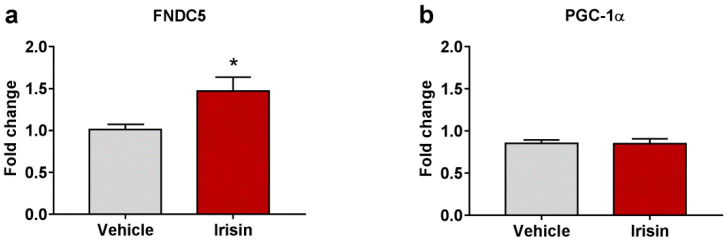
The effect of irisin treatment on FNDC5 brain expression. Gene expression of Fndc5 (**a**) and Pgc-1α (**b**) was assessed by qRT-PCR. Histograms represent mean expression ± SEM. Unpaired two-tailed Student’s *t*-test was used for statistical analysis. * *p* < 0.05.

To evaluate if irisin treatment modulated the FNDC5/irisin system in the brain, the expression of FNDC5, and its related transcriptional regulator, PGC-1α, was analyzed by quantitative real-time PCR (qRT-PCR). Results revealed that irisin injections determined a significant increase in brain FNDC5 mRNA levels compared to controls (1.471 ± 0.166 vs. 1.011 ± 0.062; * *p* < 0.05) (Figure 4a). However, irisin did not affect the expression of brain PGC-1α (0.850 ± 0.057 vs. 0.852 ± 0.042; *p* = 0.525) (Figure 4b). Table 2 reports a summary of these results.

### 2.6. Irisin Impact on Neurotrophic/Growth Factors Expression

**Figure 5:**
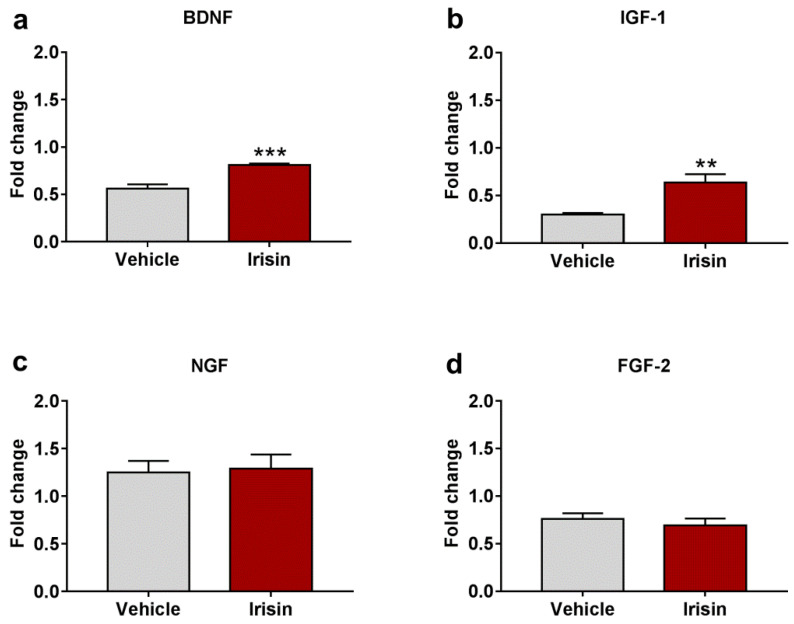
Irisin effect on neurotrophic factor expression. Gene expression of Bdnf (**a**), Igf-1 (**b**), Ngf (**c**), and Fgf-2 (**d**). Histograms represent mean expression ± SEM. Unpaired two-tailed Student’s *t*-test was used for statistical analysis. ** *p* < 0.01; *** *p* < 0.001.

In order to determine whether brain neutrophic/growth factors were regulated by irisin administration, the gene expression of BDNF, IGF-1, NGF, and FGF-2 was examined. Irisin significantly induced Bdnf gene expression compared to controls (0.810 ± 0.016 vs. 0.562 ± 0.045; *** *p* < 0.001) (Figure 5a). Moreover, IGF-1 mRNA levels were also increased in irisin-treated mice (0.638 ± 0.086 vs. 0.302 ± 0.015; ** *p* < 0.01) (Figure 5b). No differences were observed for brain NGF (1.288 ± 0.149 vs. 1.248 ± 0.123; *p* = 0.839) (Figure 5c) and FGF-2 (0.691 ± 0.073 vs. 0.761 ± 0.057; *p* = 0.464) (Figure 5d) expression between the experimental groups. Table 2 reports a summary of these results.

### 2.7. Irisin Influence on Cytokine Expression in Brain

**Figure 6:**
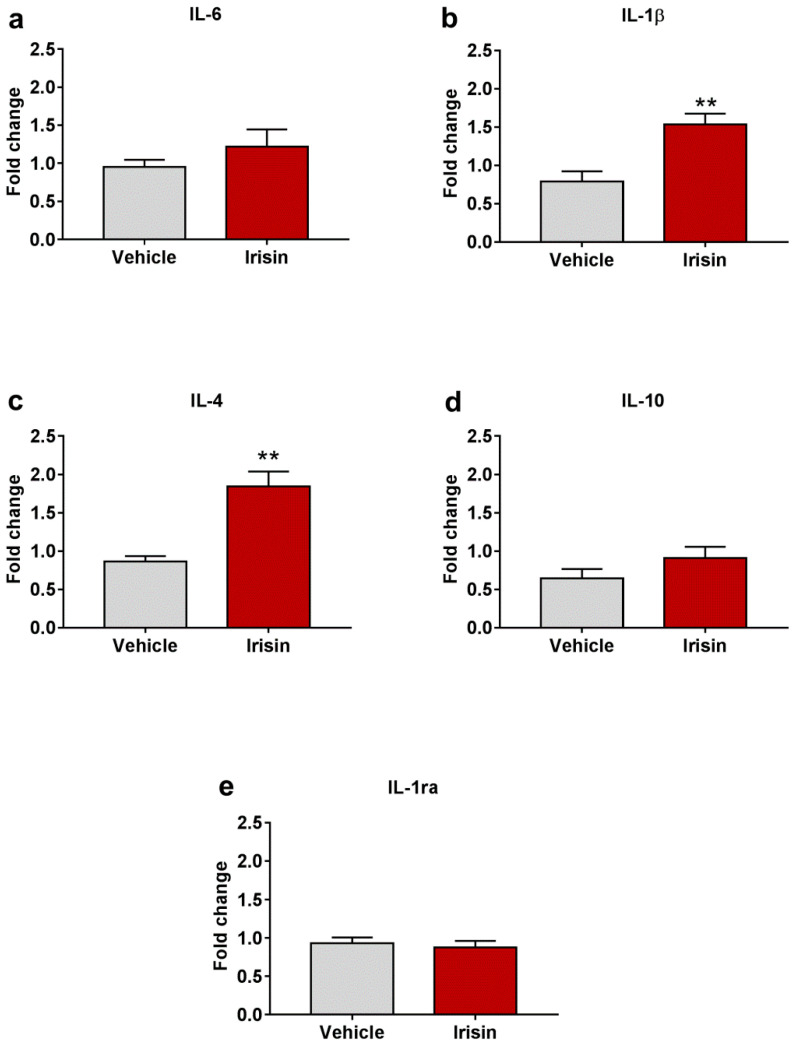
Irisin effect on cytokine profile in brain. Gene expression of Il-6 (**a**), Il-1β (**b**), Il-4 (**c**), Il-10 (**d**), and Il-1ra (**e**). Histograms represent mean expression ± SEM. Unpaired two-tailed Student’s *t*-test was used for statistical analysis. ** *p* < 0.01.

With the aim to assess the role of irisin as a modulator of the cytokine profile in brain, the gene expression of selected pro- (IL-6 and IL-1β) and anti-inflammatory (IL-4, IL-10, and IL-1ra) cytokines was evaluated. Both Il-1β (1.534 ± 0.141 vs. 0.789 ± 0.133; ** *p* < 0.01) (Figure 6b) and Il-4 (1.843 ± 0.196 vs. 0.863 ± 0.073; ** *p* < 0.01) (Figure 6c) were significantly upregulated in irisin-treated mice. A slight increase, not statistically significant, was observed for IL-6 (1.220 ± 0.224 vs. 0.950 ± 0.097; *p* = 0.267) (Figure 6a), and IL-10 (0.907 ± 0.149 vs. 0.645 ± 0.121; *p*= 0.202) (Figure 6d) mRNA levels. As far as IL-1ra is concerned, its expression was superimposable between the two groups of treatment (0.874 ± 0.087 vs. 0.931 ± 0.076; *p* = 0.630) (Figure 6e). Table 2 reports a summary of these results.

## 3. Discussion

Treatment for depression aiming to reduce both depressive manifestations and cognitive deficits preceding or following MDD onset varies according to the severity of the disease. Among the available therapies, the most effective seems to be the combination of pharmacological and psychotherapeutic interventions [31]. Even if the current pharmacological strategies involve the use of a wide range of drugs acting on different targets, the investigations on the mechanism of action of new molecules with antidepressant properties are still ongoing [32,33,34,35]. In addition to the above-mentioned therapies, non-invasive brain stimulation (NIBS) techniques, such as repetitive transcranial magnetic stimulation (rTMS), transcranial electrical stimulation (TES), and electroconvulsive therapy (ECT), acting on cortical excitability, have been proposed to obtain long-lasting effects on depressive symptoms in patients that do not respond to standard interventions [36]. NIBS were able to specifically modulate the abnormal activity of neural circuits in the amygdala, the medial prefrontal cortex and the hippocampus, and, consequently, the cognitive functions related to the memory acquisition and consolidation in depression and anxiety disorders [37,38].

In this study, the potential antidepressant role of intermittent i.p. r-irisin administration (100 µg/kg/weekly for 1 month) was evaluated by two behavioral tests commonly used to screen the efficacy of drugs conceived to treat depression disorders, i.e., the TST and the FST [39,40]. Irisin treatment significantly decreased the immobility time in the TST performed 2 h after the last irisin injection. Notably, this antidepressant effect was also observed 24 h later, as irisin-treated mice showed significantly higher mobility compared to the control group in the FST. 

Stress is listed among the risk factors for clinically significant depression [41]. As both tests exposed mice to a stress condition caused by the presence of water (FST) and unfavorable position (TST), the concentration of the stress biomarker cortisol was assayed in mouse serum. A slight reduction, not statistically significant, was noted in cortisol levels in the irisin group with respect to control mice suggesting that irisin could be involved in the stress control during the performance of the behavioral experiments. 

Considering the effect of irisin administration on cortical bone mass increase [42], the body weight analysis was performed in the experimental groups, and no significant differences were noted. In line with this observation, previous studies (unpublished data) reported overlapping results between the irisin-treated mice and controls.

To exclude the hypothesis that the young mouse hyperlocomotion could be caused by a possible psychostimulant irisin effect that may invalidate the TST and FST results, the control group and irisin-treated mice were subjected to the OFT. From the analysis of the wall rearing number, a parameter indicative of locomotor activity, it emerged that the irisin-treated mice results were overlapping with those of the control group. Taking into account the recommendations of Sturman et al. [43] to discriminate the rear behaviors in supported (wall rearing) and unsupported rearing to assess locomotion and emotional behavior, respectively, the number of unsupported rearing was also recorded. Surprisingly, a positive trend, even not statistically significant, was observed in rearing counts in treated mice suggesting a potential effect of irisin in anxiety-like behavior reduction. In accordance with this hypothesis, the mice treated with irisin also exhibited other behavioral signs of reduced anxiety, such as an increase (not statistically significant) in the time spent in the OFT arena center and the complete absence of jumping. Considering that during the behavioral tests, defecation is associated with increased anxiety [44], the fecal boli count was evaluated. Here, a not significant slight decrease in feces excretion was found in the irisin group in the FST. In the OFT, no differences were noted between the experimental groups; however, a weak negative correlation, not statistically significant, was found between the fecal boli number and the rearing count. This finding further suggests the effect of irisin in reducing anxiety. It is important to point out that depression is often associated with or caused by anxiety disorders [45]. Thus, in this regard, irisin administration could have a dual beneficial effect in reducing both depressive behaviors and anxiety. Similar to the present study, Siteneski et al. demonstrated an antidepressant as well as a potential anxiolytic role of irisin in mice after i.c.v. injections [28].

Molecular and behavioral studies deepened the role of neurotrophic/growth factors (such as BDNF, NGF, IGF-1, and FGF-2) in depression, demonstrating that the levels of those factors were reduced in animal models and in depressed and/or stressed patients (“neurotrophic hypothesis of depression”) [46]. Since the neurotrophic factors play a pivotal role in neuron maintenance and survival in the adult brain, their reduction in depressed/stressed subjects induced brain structural alterations, specifically neuronal atrophy and reduced neurogenesis in the hippocampus [47,48]. Among neurotrophins, BDNF is one of the most highly studied due to its involvement in neurogenesis and neuronal plasticity; for these features, different types of antidepressant drugs act to increase its expression [47]. Notably, in this study, Bdnf gene expression was significantly increased by irisin long-term administration. Likewise, Wrann et al. found the Bdnf up-regulation in the brain after the peripheral delivery of irisin precursor FNDC5 through an adenoviral vector as well as following a program of physical exercise [25]. In addition to BDNF, also IGF-1, FGF-2, and NGF have been implicated in depressive disorders [47]. IGF-1 is a growth factor involved in the expression of BDNF, and it acts in several brain processes, including synaptic plasticity, neurogenesis in the adult brain, and neuron differentiation [49]. IGF-1 decrease induced clinical alterations in MDD, such as cognitive dysfunctions [50]. Like BDNF, here, a significant increase was observed for Igf-1 gene expression in irisin-treated mice, while no differences were found for Ngf and Fgf-2. Interestingly, the antidepressant effect of irisin seemed to be principally associated with two neurotrophins (BDNF and IGF-1), whose combined effect on depression reduction has already been reported in rats by i.c.v. irisin injections [51]. 

Taking into account the crosstalk between the CNS and the immune cells, at the beginning of the 1990s, the “cytokine theory of depression” was formulated, proposing the involvement of proinflammatory cytokines in disease pathogenesis [52]. Indeed, accumulating evidence showed an increase in proinflammatory mediators, principally IL-6, IL-1β, interferon-γ (IFN-γ), and TNF-α in depressed individuals and in patients developing depressive symptoms after therapeutic treatments, such as IFN-α therapy for chronic hepatitis C [53,54,55,56,57]. In line with these findings, it has been reported that the serum concentration of some proinflammatory cytokines was further increased in the severe form of depression [58]. Additionally, behavioral variations similar to those observed in depressed patients (i.e., decreased activity, anhedonia, cognitive dysfunction, and sleep quality alterations) were found in animal models with an increased proinflammatory profile [59]. Recently, the role of anti-inflammatory cytokines in attenuating the effect of inflammatory mediators (i.e.*,* IL-10, IL-4, and IL-1ra) has also been taken into account in depression studies [53]. A regular decrease in IL-10 was detected along with depressive symptoms increase [58]. Park and co-workers described the positive impact of IL-4 in the reduction of depressive-like behaviors that were induced in rats by i.c.v. IL-1β injection [60]. Here, the analysis of the effect of irisin administration on selected genes encoding for pro- and anti-inflammatory cytokines showed that IL-1β and IL-4 mRNA levels were both increased in irisin-treated mice, while a positive trend, not statistically significant, was found for IL-6 and IL-10. As far as IL-1ra is concerned, the expression of this endogenous antagonist of the IL-1 pathway was not modulated by irisin injections. These data suggest that although irisin upregulated the expression of inflammatory mediators, it was also able to increase the expression of their antagonists. It should be specified that cytokines are a group of factors with pleiotropic effects, and their impact on the CNS is deeply affected by microenvironmental factors, the in situ concentration, etc. [61]. Moreover, the existence of a complex interplay among cytokines that makes it difficult to understand their individual effects is well known [62]. In light of this evidence, further studies will be necessary to elucidate the effect of irisin in the modulation of the inflammatory state in the CNS also through the use of suitable animal models.

Collectively, it is possible to assume that systemic irisin can cross the blood–brain barrier exerting antidepressant-like effects and displaying a possible anxiolytic role. However, although PGC-1α expression did not change after irisin administration, a significant increase in the expression of its precursor FNDC5 was found only in irisin-treated mice. Thus, here it is not possible to exclude that the irisin beneficial effects on depressive behaviors were also a consequence of its endogenous production.

## 4. Materials and Methods

### 4.1. Animals 

The present study used 18 male black Swiss mice (C57BL/6) aged 12–13 weeks, weighing 24–31 g, and housed in standard rodent polypropylene cages with free access to food and tap water ad libitum. The body weight was noted before each injection in the experimental groups. Controlled noise, lighting (12/12 h light/dark cycle, 6 a.m.–6 p.m.), and temperature (21 ± 1 °C) conditions were maintained. Ten days before the start of the experimental protocol, animals were separated and kept in groups of 3 or 4. Experiments were carried out in the light phase, between 12:00 p.m. and 5:00 p.m. 

### 4.2. Irisin Treatment

Mice were treated with an intraperitoneal injection (i.p.) of a vehicle (physiologic water sterilized by 0.22 μ filtration) (*n* = 9) or r-irisin (100 µg/kg/weekly) (*n* = 9) for 28 days and sacrificed at the end of treatment, 24 h after the 5th injection. r-Irisin was provided by Adipogen International (San Diego, CA, USA). Irisin concentration was chosen based on prior studies conducted by our research group [42].

### 4.3. Behavioral Tests

All behavioral paradigms, Tail Suspension Test (TST), Forced Swim Test (FST), and Open Field Test (OFT), were conducted between 12:00 a.m. and 5:00 p.m. by the same researcher. Mice were brought into the experiment room 30 min before testing to allow them to acclimate. OFT was carried out 1 h after vehicle or irisin administration. The TST and FST were performed 2 and 24 h after the last treatment (5th inj), respectively. 

#### 4.3.1. Forced Swim Test (FST)

This test was used to evaluate mouse depressive-like behavior and despair in stressful situations following the protocol described by Porsolt et al. and adapted for experiments in mice [40,63]. In brief, mice were individually placed in a transparent acrylic cylinder (13 cm diameter × 24.5 cm high) filled with tap water (19 cm depth) at 25 ± 1 °C for 6 min. Each session consisted of 2 min of training (pre-test) followed by 4 min of testing. However, total time spent in immobility (i.e., a floating position without climbing or swimming) was evaluated during the last 4 min of testing. The number of fecal boli expelled was also noted as an indicator of emotionality [64].

#### 4.3.2. Tail Suspension Test (TST)

In this test, frequently used to study depressive-like behavior, mice were subjected to a short-term stress state caused by the tail suspension. According to the protocol described by Steru L et al. [39], mice were individually suspended by the tail at 25 cm above the floor level. Adhesive tape was placed at the end of the tail to maintain mice in a position in which they could not escape or climb. The murine activity was recorded for a period of 6 min. Mice were considered immobile when they displayed inactivity with a complete lack of movements.

#### 4.3.3. Open Field Test (OFT)

OFT was performed to assess mouse locomotor activity and/or anxiety-related behaviors, using the protocol described by Seibenhener and Wooten [65]. The apparatus consisted of an empty translucent acrylic square arena (40 × 40 × 40 cm) with a semi-transparent bottom and high walls that prevented mouse escape. Animals were placed in the center of the arena, and the number of rearing and wall-rearing (unsupported and supported vertical exploration, respectively), total time spent in the inner zone, jumping number, and fecal boli count were evaluated during a 10-minute observation.

### 4.4. Cortisol Determination

Mouse blood samples (about 1 mL) were collected into ice-cooled centrifugal tubes after the performance of the last behavioral test (TST). Blood samples were centrifuged (1700× *g*, 10 min, 4 °C) to separate serum. The serum samples were stored at −80 °C until the cortisol assay was performed. The cortisol concentration in the mouse serum was measured by a competitive enzyme immunoassay kit (Abcam, Cambridge, UK, Cat. No. ab285260) according to the manufacturer’s instructions. Each sample was assayed in triplicate.

### 4.5. RNA Extraction, cDNA Synthesis, and qRT-PCR Assays on Brain Tissues

Brains were dissected from the mice after the performance of the TST. Total RNA was extracted from brain tissues by spin columns (RNeasy Mini Kit, Qiagen, Hilden, Germany). Then, 1 μg of RNA was reverse transcribed in a 20 μL reaction volume using iScript Reverse Transcription Supermix (Bio-Rad Laboratories, Hercules, CA, USA) in a thermal cycler (My cycler; Bio-Rad Laboratories). Subsequently, qRT-PCR assays were performed with SsoFast EvaGreen Supermix (Bio-Rad Laboratories, Hercules, CA, USA) on a CFX96 Real-Time System (Bio-Rad Laboratories) for 40 cycles (denaturation 95 °C for 5 s; annealing/extension 60 °C for 10 s) after an initial step (30 s) for enzyme activation at 95 °C. Primer Blast (https://www.ncbi.nlm.nih.gov/tools/primer-blast/, (accessed on 20 April 2022) was used to design all primers. Table 3 reported the details of oligonucleotide sequences for target and reference genes. In each assay, the cDNAs of both reference genes and each gene of interest were measured simultaneously under identical conditions. The specificity of the PCR reactions was confirmed by the analysis of the melt curve: for each amplicon, the detected melting temperature was the expected one. Each sample was assayed in triplicate, and quantitative analysis was obtained using the delta–delta threshold cycle (ΔΔCT) method and expressed as a fold change compared to control.

### 4.6. Data Processing and Statistical Analyses

Statistical analyses were performed by using the GraphPad Prism statistical software release 7.0 (GraphPad Software, San Diego, CA, USA). Datasets were initially checked for normal distribution using Shapiro–Wilk normality test. The comparison of the body weights among the experimental groups at different time points was executed by one-way ANOVA. Statistical significances of differences between vehicle or r-irisin-treated mice were assessed using Student’s t or Mann–Whitney test. The Spearman’s rank correlation test was used to determine the association between fecal boli and rearing counts in the OFT. Values of *p* < 0.05 were considered statistically significant.

## 5. Conclusions

To the best of our knowledge, this is the first study evidencing that intermittent irisin i.p. administration is able to induce an antidepressant-like behavior persisting up to 24 h after irisin injection. The OFT results suggested that irisin may also have an anxiolytic-like action. These effects could probably involve the modulation of endogenous brain factors such as neurotrophic/growth factors (BDNF and IGF-1) and cytokines (IL-1β, IL-4, IL-6, and IL-10). Overall, the results reported in the present work confirm previous findings regarding the antidepressant effect of irisin in mice. However, with respect to other studies using i.c.v. injections, here it was demonstrated that the irisin antidepressant effect may be observed even when the myokine is administrated by systemic injection. This could pave the way toward intriguing preclinical research in humans.

## 6. Limitations and Future Directions

In some previous studies, the antidepressant effect of drugs was investigated using animal models of depression induced by a sequence of unpredictable stressors (chronic unpredictable stress), genetic modifications, and the chronic administration of a wide range of different molecules [27,66]. In the present work, the effect of systemic intermittent irisin administration was demonstrated in healthy young mice. Therefore, to overcome this limitation, it is desirable that further studies are performed on suitable animal models. Moreover, it would be interesting to evaluate the effect of irisin administration on the mammalian target of the rapamycin (mTOR)-signaling pathway that plays a key role in the preclinical tests of novel antidepressant drugs [67]. Finally, further studies on aged animal models will be needed to focus on the interplay among MDD, dementia, and neurodegenerative diseases, as depression is not only the most frequent noncognitive disorder associated with cognitive deficits but also a risk factor that contributes to worsening the neurodegeneration [68].

## Figures and Tables

**Table 1:** Summary of behavioral test results.

Test	Parameter	Vehicle-Treated Mice (Mean ± SEM)	Irisin-Treated Mice (Mean ± SEM)	*p*-Value
TST	Immobility (s)	217.80 ± 11.88	163.80 ± 14	* *p* < 0.05
FST	Immobility (s)	162.33 ± 7.409	133.44 ± 10.39	* *p* < 0.05
	Fecal boli count	5.33 ± 0.866	4 ± 1.041	*p* = 0.339
OFT	Wall rearing count	21.75 ± 3.35	22 ± 1.414	*p* = 0.9373
	Rearing count	17 ± 3.769	23.25 ± 1.75	*p* = 0.1211
	Fecal boli count	1.25 ± 0.4532	1.333 ± 0.2887	*p* = 0.8283
	Time in inner zone (s)	30 ± 8.426	45.42 ± 11.7	*p* = 0.3472
	Jumping count	3.25 ± 2.358	0 ± 0	*p* = 0.1333

* *p* < 0.05.

**Table 2:** Summary of qRT-PCR results in brain.

Gene Expression	Fold Change (Mean ± SEM)	Fold Change (Mean ± SEM)	*p*-Value
FNDC5	1.011 ± 0.062	1.471 ± 0.166	* *p* < 0.05
PGC-1α	0.852 ± 0.042	0.850 ± 0.057	*p* = 0.525
BDNF	0.562 ± 0.045	0.810 ± 0.016	*** *p* < 0.001
IGF-1	0.302 ± 0.015	0.638 ± 0.086	** *p* < 0.01
NGF	1.248 ± 0.123	1.288 ± 0.149	*p* = 0.839
FGF-2	0.761 ± 0.057	0.691 ± 0.073	*p* = 0.464
IL-6	0.950 ± 0.097	1.220 ± 0.224	*p* = 0.267
IL-1β	0.789 ± 0.133	1.534 ± 0.141	** *p* < 0.01
IL-4	0.863 ± 0.073	1.843 ± 0.196	** *p* < 0.01
IL-10	0.645 ± 0.121	0.907 ± 0.149	*p* = 0.202
IL-1ra	0.931 ± 0.076	0.874 ± 0.087	*p* = 0.630

* *p* < 0.05; ** *p* < 0.01; *** *p* < 0.001.

**Table 3:** Primer sequences used for quantitative real-time PCR.

Gene Name	Gene Bank Number	Primer sequence (5′-3′)	Product Size (bp)	Annealing Temperature (°C)
Gapdh	NM_001289726.1	Forward ACACCAGTAGACTCCACGACA Reverse ACGGCAAATTCAACGGCACAG	145	60.48 62.59
Fndc5	NM_027402.4	Forward GTGCTGATCATTGTTGTGGTCC Reverse ATCATATCTTGCTGCGGAGGAG	169	60.10 60.03
Pgc-1α	NM_008904.3	Forward CCCTGCCATTGTTAAGACC Reverse TGCTGCTGTTCCTGTTTTC	161	55.87 56.35
Bdnf	NM_001048139.1	Forward TGAAGTTGGCTTCCTAGCGG Reverse CCTGGTGGAACTTCTTTGCG	146	60.04 59.41
Fgf-2	NM_008006.2	Forward GCTGCTGGCTTCTAAGTGTG Reverse GTCCAGGTCCCGTTTTGGAT	158	59.20 59.96
Igf-1	NM_001111276.1	Forward TGCCTGGGTGTCCAAATGTA Reverse TGTATCTTTATTGCAGGTGCGG	170	59.23 59.06
Ngf	NM_001112698.2	Forward GGAGCGCATCGAGTTTTGG Reverse CCTCACTGCGGCCAGTATAG	136	59.57 59.97
Il-4	NM_021283.2	Forward TCACAGCAACGAAGAACACCA Reverse CAGGCATCGAAAAGCCCGAA	158	60.41 61.31
Il-10	NM_010548.2	Forward GTAGAAGTGATGCCCCAGGC Reverse CACCTTGGTCTTGGAGCTTATT	187	60.46 58.31
Il-1ra	NM_001039701.3	Forward GTGGCCTCGGGATGGAAAT Reverse TGGTTAGTATCCCAGATTCTGAAGG	148	59.77 59.63
Il-6	NM_001314054.1	Forward CCAAGAGATAAGCTGGAGTCACA Reverse CGCACTAGGTTTGCCGAGTA	121	59.80 60.11
Il-1β	NM_008361.4	Forward TGCCACCTTTTGACAGTGATG Reverse ATGTGCTGCTGCGAGATTTG	136	59.04 59.55

## Data Availability

Not applicable.

## Supplementary Materials

The following supporting information can be downloaded at: www.mdpi.com/article/10.3390/ijms23147596/s1.
