# Supplementary material for: Antidepressant Effect of Intermittent Long-Term Systemic Administration of Irisin in Mice
Source: Int J Mol Sci. 2022 Jul 8;23(14):7596. doi: 10.3390/ijms23147596 (PMC9320584; doi:10.3390/ijms23147596)
Supplement: Supplementary file 1 [file ijms-23-07596-s001.zip › ijms-1776716-supplementary.pdf]

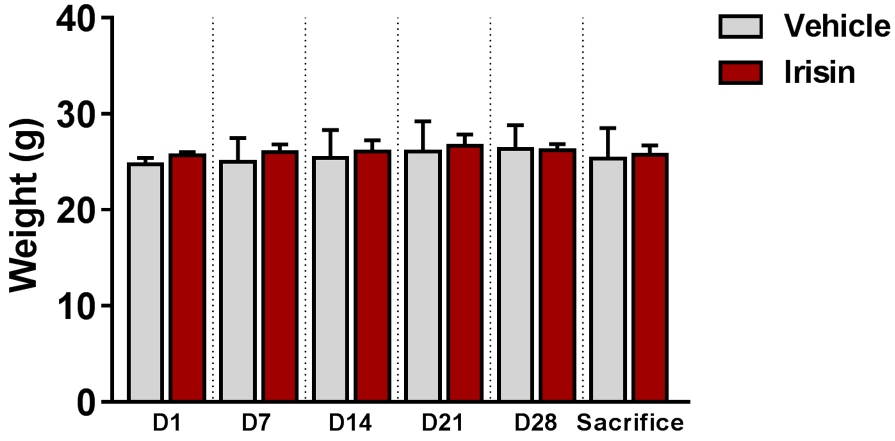

Figure S1. Irisin effect on body weight at day 1 (D1), day 7 (D7), day 14 (D14), day 21 (D21), day 28 (D28) and before mouse sacrifice. Histograms represent mean values ± SEM. One-way ANOVA was used for statistical analysis.
